# Genetic modification of Bluetongue virus by uptake of "synthetic" genome segments

**DOI:** 10.1186/1743-422X-7-261

**Published:** 2010-10-07

**Authors:** René GP van Gennip, Daniel Veldman, Sandra GP van de Water, Piet A van Rijn

**Affiliations:** 1 Central Veterinary Institute of Wageningen UR (CVI) Department of Virology, P.O. Box 65, 8200 AB Lelystad, The Netherlands

## Abstract

Since 1998, several serotypes of Bluetongue virus (BTV) have invaded several southern European countries. In 2006, the unknown BTV serotype 8 (BTV8/net06) unexpectedly invaded North-West Europe and has resulted in the largest BT-outbreak ever recorded. More recently, in 2008 BTV serotype 6 was reported in the Netherlands and Germany. This virus, BTV6/net08, is closely related to modified-live vaccine virus serotype 6, except for genome segment S10. This genome segment is closer related to that of vaccine virus serotype 2, and therefore BTV6/net08 is considered as a result of reassortment. Research on orbiviruses has been hampered by the lack of a genetic modification method. Recently, reverse genetics has been developed for BTV based on ten in vitro synthesized genomic RNAs. Here, we describe a targeted single-gene modification system for BTV based on the uptake of a single in vitro synthesized viral positive-stranded RNA. cDNAs corresponding to BTV8/net06 genome segments S7 and S10 were obtained by gene synthesis and cloned downstream of the T7 RNA-polymerase promoter and upstream of a unique site for a restriction enzyme at the 3'-terminus for run-off transcription. Monolayers of BSR cells were infected by BTV6/net08, and subsequently transfected with purified in vitro synthesized, capped positive-stranded S7 or S10 RNA from BTV8/net06 origin. "Synthetic" reassortants were rescued by endpoint dilutions, and identified by serotype-specific PCR-assays for segment 2, and serogroup-specific PCRs followed by restriction enzyme analysis or sequencing for S7 and S10 segments. The targeted single-gene modification system can also be used to study functions of viral proteins by uptake of mutated genome segments. This method is also useful to generate mutant orbiviruses for other serogroups of the genus *Orbivirus *for which reverse genetics has not been developed yet.

## Findings

Bluetongue (BT) is an arthropod-borne disease; transmission to ruminants, including cattle, sheep, and goats, occurs by bites of species of *Culicoides*. Bluetongue is listed as a 'notifiable disease' by the Office International des Epizooties (OIE) [1] causing severe hemorrhagic disease with fever, lameness, coronitis, swelling of the head (particularly the lips and tongue) and death. Bluetongue virus (BTV) belongs to the family *Reoviridae*, genus *Orbivirus *[2].

The genome of BTV consists of ten linear double-stranded RNA genome segments encoding the seven structural proteins VP1 to VP7, and three nonstructural proteins, NS1, NS2 and NS3/NS3a [3-7]. The two inner layers of the BTV particle, identified as the 'sub-core' and 'core', are composed of major structural proteins VP3 and VP7, and are encoded by genome segment S3 and S7. The innermost shell, the 'subcore' consists of VP3 and surrounds one copy of each of the ten genome segments and the three enzymatic structural proteins VP1, VP4 and VP6, which are encoded by S1, S4 and S9, respectively.

Since 1998, BTV serotypes 1, 2, 4, 9, and 16 have invaded European countries around the Mediterranean Basin. The outbreak by BTV8/net06 (sample nr. BTV-8 NET2006/04 in the dsRNA virus reference collection (dsRNA-VRC) at IAH Pirbright, [8]) starting in August 2006 [9] has resulted in the largest BT-outbreak ever recorded. More recently, BTV6/net08 (sample BTV-6 NET2008/05 in the dsRNA-VRC at IAH Pirbright, [10]) was reported in The Netherlands [11] and Germany [12] in 2008. BTV6/net08 is closely related to modified-live vaccine virus serotype 6, but genome segment S10 showed the highest homology (98.4%) with that of vaccine virus serotype 2 (RSAvvv2/02 in dsRNA-VRC). This suggested a reassortment between vaccine viruses serotype 6 and serotype 2 resulting in BTV6/net08. Maan et al. also suggested that BTV6/net08 was in the process of reassorting with BTV8/net06, since the blood of a PCR-positive cow contained two different S7 sequences, one of which (from the BTV6 vaccine) was selected during virus isolation in cell-culture [10]. The other S7 sequence (from the Northern field strain BTV8/06) was predominantly found in blood of this cow.

Research on BTV, including research on reassortment, has already a long scientific record (reviewed by Roy 2005; [13]). Recently, a reverse genetics system for BTV has been developed [14], and has been demonstrated to be useful to generate mutants of BTV by genetic manipulation of one or more of genome segments [15]. This system needs, however, a set of ten complete cDNAs of genome segments to rescue bluetongue virus from T7 derived RNA transcripts. Here, we describe a targeted single-gene genetic modification system as an alternative method for genetic modification of orbiviruses. This system is based on the uptake of one *in vitro *synthesized viral RNA in an ongoing infection. We have focused on the uptake of genome segments S7 or S10 originating from BTV8/net06 in BTV6/net08, although the method is proposed to be broadly applicable for all genome segments and all orbiviruses.

**Figure 1:**
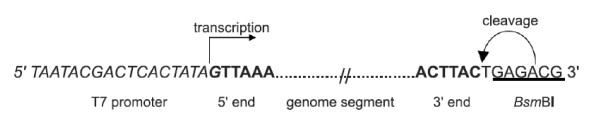
**Schematic overview of plasmids containing the full-length BTV genome segment**. A full-length BTV genome segment flanked by a T7 promoter and a BsmBI (for S10) or BbsI (for S7) restriction enzyme site which defines the BTV 3'end sequence during transcription. The nucleotides of the ultimate 5'- and 3'-ends of the BTV genome segment are presented in bold symbols. The sequence of the T7 promoter is italicized, and the BsmBI site is underlined. The positions of the start of transcription and digestion by restriction enzymes for run-off transcription are indicated by arrows.

Genome segments S7 and S10 were synthesized by Genscript Corporation (Piscataway, NJ) based on the identical sequences AM498057.2 and FJ183380.1 for S7, and the identical sequences AM498060.1 and FJ183383.1 for S10 of Genbank. cDNAs were cloned in plasmid pUC57 under control of the T7 RNA-polymerase promoter and a site for a restriction enzyme at the 3'-terminus for defined run-off transcription (Figure 1, depicted from Boyce et al., [14]). Plasmids were maintained in *E. coli *DH5α, and were purified using QIAfilter Plasmid Midi Kit (Qiagen).

Plasmid DNA was digested with *Bbs*I for S7 or with *BsMB*I for S10, and was purified by standard procedures. One μg of digested plasmid DNA was used for *in vitro *RNA run-off transcription with 5' cap analogue using the MESSAGE mMACHINE T7 Ultra Kit (Ambion). In this reaction, a ratio of 4:1 of anti-reverse cap analogue to rGTP was used. Synthesized RNA was cleaned by use of MEGAclear columns (Ambion) according to the manufacturer's instructions, and eluted RNA was stored at -80°C.

Monolayers of 10^5 ^BSR cells ([16], gift of P. Roy) were infected at a multiplicity of infection (moi) of 0.1 with BTV6/net08, which has been isolated on embryonated eggs (e1), followed by three passages on BHK21 cells (bhk3), and two passages on BSR cells (bsr2) (BTV6/net08/e1/bhk3/bsr2). At one hr post infection (hpi), infected monolayers were transfected with 400 ng synthesized RNA transcripts of S7 or S10 using 1 μl lipofectamine™2000 (1:2.5; 1 mg/ml Invitrogen) in Opti-MEM^® ^I Reduced Serum Medium according to manufacturer's conditions for 4 hrs, after which it was refreshed with 1 ml of Dulbecco's Modified Eagle Medium (DMEM) supplemented with 5% FBS and 1% of Penicillin/Streptomycin/Fungizone. At 40 hpi, supernatants were harvested, and virus was cloned by endpoint dilution in M96-wells on BSR cells. At 3 days post infection (dpi), supernatants were collected from wells with cells developing cytopathogenic effect (CPE).

Infection of the respective monolayers was confirmed by immunostaining with monoclonal antibody (Mab) produced by ATCC-CRL-1875 directed against VP7 (data not shown). Typically, viruses in 48 supernatants were multiplied in M24 wells in BSR cells by adding 75 μl supernatant in 1 ml of DMEM supplemented with 5% FBS and 1% of Penicillin/Streptomycin/Fungizone. After development of CPE, 2-3 dpi, supernatants were collected and stored at -80°C. Viral RNA was isolated from 200 μl of supernatant with the High Pure Viral RNA kit (Roche).

A serogroup-specific duplex RT-PCR was used for amplification of genome segment S7 [17]. For partial amplification of genome segment S10, the in-house developed serogroup-specific diagnostic RT-PCR-assay was used [18]. Differentiation between both segments S7 and segments S10 was performed by either restriction analysis or sequencing of amplicons. For S7, 5 μl of the RT-PCR reaction was digested with *Pst*I and *Bgl*II and analyzed by agarose gel electrophoresis. For S10, gel-purified amplicons were sequenced using the BigDye^® ^Terminator v3.1 Cycle Sequencing Kit (Applied Biosystems, Foster City, IA, USA) in a ABI PRISM^® ^3130 Genetic Analyzer (Applied Biosystems, Foster City, IA, USA). In order to genetically serotype the cloned viruses, in-house developed serotype-specific PCR-assays for serotypes 6 and 8, based on segment S2 of BTV, were carried out using LightCycler RNA Master Hybridization Probes kit and a LightCycler 2.0 PCR machine (both supplied by Roche Diagnostics, Almere, Netherlands). For the BTV6-S2 serotype-specific RT-PCR forward primer 5'-AGGAACAGTCGGCTTATCAC-3', reverse primer 5'-TTCGCTAATGTGCTTCTCCAT-3' (Eurogentec b.v., Maastricht, Netherlands) and taqmanprobe 5'-6FAM- TTGTCAGCTTTACGCAAACCCCG-BHQ-3' (Tib MolBiol, Berlin, Germany) were used. For the BTV8-S2 serotype-specific RT-PCR forward primer 5'-CGGAGACAGCGCAGTATGTA-3', reverse primer 5'-CCTCGGTAGTATCCCTCACG-3' (Eurogentec b.v., Maastricht, Netherlands) and taqmanprobe 5'-6FAM-ACATACGATGCCYTCGGAGGATTCTG-BHQ-3' (Tib MolBiol, Berlin, Germany) were used. Template RNA (5 μl) was added to a reaction mixture containing 0.25 μM of the forward and reverse primer, 0.25 μM probe, 2.75 mM MnCl2, 7.5 μl LightCycler mix and 0.2 μl RNAsin (RNAsin, 40 U/μl, Promega Benelux b.v., Leiden, Netherlands) in a final volume of 20 μl. Thermocycling conditions of the RT-PCR were: 20 s 98°C, 20 min 61°C, 30 s 95°C (1 s 95°C, 10 s 61°C, 15 s 72°C) × 45 cycles followed by 30 s 40°C and storage at 4°C. Amplification was monitored real-time by OD530/OD640 using LightCycler software version 4.05 (Roche Diagnostics b.v., Almere, Netherlands).

**Figure 2:**
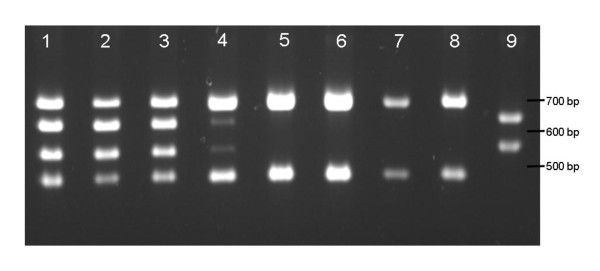
**Restriction enzyme analysis of amplicons derived from S7 of different passages of a mixture of reassortant and parental virus**. Amplicons were digested with BglII and PstI. Segment S7 of BTV8 (S7^8^) digested with PstI (unique for S7^8^) results in fragments of 471 and 685 bps (see lane 8), whereas segment S7 of BTV6 (S7^6^) digested with BglII (unique for S7^6^) results in fragments of 536 and 620 base pairs (bps) (see lane 9). Several blind passages (p) of the initial mixture of reassortant BTV6/net08/S7^8^ and parental virus BTV6/net08 were analyzed by digestion with both restriction enzymes; p1 (lane 1), p2 (lane 2), p4 (lane 3), p5 (lane 4), and p6 (lane 5). Passage 6 was cloned by end point dilution and two finally cloned reassortants BTV6/net08/S7^8^ were passed twice and analyzed (lanes 6 and 7). Analysis of amplicons derived from segment S7 of BTV8/net06 and parental virus BTV6/net08 are presented in lanes 8 and 9, respectively.

For segment S7, 1 out of 30 cloned viruses contained S7 originating from BTV8/net06 (i.e BTV6/Net08/S7^8^). This finding was based on both positive and negative differentiation; the presence of a *Pst*I site in S7 of BTV8, and the absence of a *Bgl*II site in case of S7 of BTV6 (Table 1, and Figure 2, lane 8 and 9). Furthermore, the sequence of this segment S7 was 100% identical to S7 of BTV8/net06. This cloned virus was genetically serotyped as serotype 6, whereas no detectable signal for serotype 8 was present (Table 1). The unique combination of S2 of BTV6 and S7 originating from BTV8 clearly proves the presence of "synthetic" reassortant virus BTV6/net08/S7^8 ^(Table 1).

**Table 1:** Characterization of reassortant viruses.

virus	Genotyping on S7 **amplicon**^**a**^	Genotyping on S10 **amplicon**^**b**^	BTV6 serotype **specific PCR**^**c**^	BTV8 serotype **specific PCR**^**d**^
BTV8/net06	8	8	-	+
BTV6/net08	6	6	+	-
BTV6/net08/S7^8^	8	6	+	-
BTV6/net08/S10^8^	6	8	+	-

a. S7 amplicons were digested with BglII and PstI and compared to that of the parental strains, see also figure 2, lanes 8 and 9. b. S10 amplicons were sequenced and compared to sequences of parental strains BTV8/net06 and BTV6/net08. Genetic serotyping by serotype-specific real-time PCR-assays was performed for serotypes 6 (c) and 8 (d). Presence or absence of a Cp-value was interpreted as + and -, respectively.

For genome segment S10, 1 out of 24 tested clones contained S10 originating from BTV8/net06 (i.e. BTV6/Net08/S10^8^) based on nucleotide differences on several positions in the amplicon. Again, the presence and absence of S2 of respectively serotype 6 and 8 was confirmed (Table 1). The "synthetic" reassortant BTV6/net08/S10^8 ^also represents a unique combination of genome segments in one BTV not seen before.

**Figure 3:**
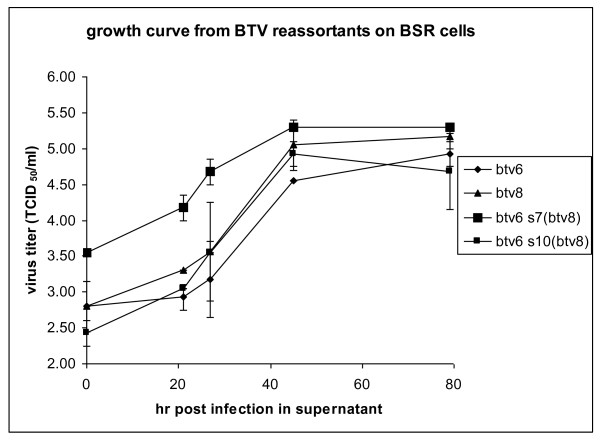
**Growth curve of parental and reassortant BTVs**. BSR monolayers were infected in duplicate by reassortant viruses BTV6/net08/S7^8^, BTV6/net08/S10^8^ and parental virus BTV6/net08 and BTV8/net06 with 0.1 moi. At 0, 21, 27, 45 and 79 hours post infection, samples of 1 ml were taken. The virus titer in collected samples were determined by end-point dilution.

In one occasion, we have also observed a mixture of both segments S7 in candidate reassortant viruses (Figure 2, lane 1). After six sequential and blind passages on BSR cells, a virus stock was obtained containing a majority S7 derived from BTV8 (Figure 2, lane 5). After cloning by end-point dilution, only reassortant BTV6 with S7 of BTV8, BTV6/net08/S7^8^, was found (Figure 2, lane 6 and 7). Enrichment of this *in vitro *rescued reassortant BTV after passaging suggests that this reassortant benefits from S7 of BTV8. This is in agreement with previous findings [10] in which also a positive selection was suggested for the reassortant BTV6 with segment S7 delivered by BTV8/net06. In order to study whether reassortant viruses BTV6/net08/S7^8 ^and BTV6/net08/S10^8 ^differ in growth characteristics, we determined growth curves on BSR cells. Therefore, confluent monolayers of BSR cells in M24-well were infected at a moi of 0.1 with BTV6/net08(e1/bhk3/bsr2), BTV8/net06(e1/bhk3), BTV6/net08/S7^8^(bsr2) and BTV6/net08/S10^8^(bsr2). After attachment to cells for 1.5 h at 37°C, supernatant was removed and stored at -80°C (t = 0). One ml of fresh DMEM with 5% FBS, 1% Penicillin/Streptomycin/Fungizone was added to the monolayers and incubation was continued. At 21, 27, 45 and 79 hours post infection (hpi), samples of the supernatants were harvested and stored at -80°C. Virus titers were determined by end-point dilution. The observed differences in virus titer at 0 hpi, which was approximately 10-fold higher for BTV6/net08/S7^8 ^(Figure 3), reflect the amount of non-attached virus. Starting from 21 hpi, virus titers in supernatants were determined reflecting the production of virus. In all samples of the growth curve, samples of BTV6/net08/S7^8 ^contained a significant higher virus titer, but the difference at the final sampling point (79 hpi) was minimal. Since no great differences in the slopes of the different growth curves were detected, the observed enrichment of this reassortant by passaging (6 times) of a mixture of BTV6/Net08 and BTV6/net08/S7^8 ^could be the result of factors other than replication and remains unclear.

Despite optimizing the uptake of an exogenous genomic RNA-segment, the here described method to generate reassortants of bluetongue virus is not very efficient. The percentage of rescued reassortant virus is approximately 3-5% for genome segments S7 and S10. However, the method is relatively easy to perform, and mass screening of reassortant candidates can be easily performed depending on the targeted gene and available tools, like discriminating Mabs and/or discriminating PCR-assays. Particularly, this method is of interest for research focusing on one genome segment, since a full set of ten cDNAs encoding complete genome segments is not required. Boyce et al [19] have developed a method with a similar aim by mixing authentic core-derived transcripts isolated from infected cells and plasmid-derived T7 transcript of which the efficiency was 15-80% to recover reassortant infectious BTV. This efficiency is significantly higher than of the method described here, but isolation and purification of intact core-derived RNAs needs a lot of preparation.

The major drawback of the here described method is the high percentage of parental virus not reassorting with delivered *in vitro *synthesized RNA. On one hand, the method could be significantly improved by reducing this virus background with discriminating specific siRNAs. Very strong reduction of virus growth has been published for African horse sickness virus, another serogroup of the genus O*rbivirus *[20]. On the other hand, the amount of *in vitro *synthesized RNA in infected cells could be further increased to improve the efficiency to rescue reassortants. This could be achieved by *in vivo *RNA synthesis by T7 RNA-polymerase expressing BSR cells after transfection of plasmids containing cDNA of a genome segment flanked by the T7 promoter and a functional ribozyme sequence. Alternatively, repeated transfection of *in vitro *synthesized RNA could increase the presence of RNA in the BTV-infected cell. Using reverse genetics, recently Matsuo et al. have shown that repeated transfection of BTV transcripts strongly improve the recovery of infectious BTV [21]. This suggests a short half-life of transfected BTV-RNAs. Thus, timing of RNA-delivery could be crucial for our method, and can also be solved by the suggested repeated RNA transfection or constitutive transcription of BTV-RNA to increase the percentage of reassortants. Summarizing, although this method is successful, we believe that this method can be significantly improved to rescue reassortant orbiviruses.

Likely, the first event, the uptake of the transfected RNA by the replicating virus is a random process. This makes this method also suitable for rescue of reassortants with other genome segments. For instance to generate reassortant virus with a different serotype by uptake of RNA of genome segment S2. For this special case, neutralizing sera or neutralizing Mabs could be used to further reduce the background of parental virus and to screen for reassortant virus.

The developed method results in the uptake by replicating BTV of RNA that was synthesized *in vitro *with cDNA as template. This opens the opportunity to use this method as genetic modification system for BTV by uptake of mutated genome segments to study viral proteins. However, we realize that rescue of mutant BTVs with a lower fitness will be more difficult. Presumably, significant improvement of the method is necessary for this purpose by either lowering the virus background, increase the chance on uptake of synthesized mutant RNA, or both. However, the opposite was not seen, reassortant BTV6/08/S7^8 ^was rescued with a similar efficiency, although this reassortant multiplies to a higher virus titer than the parental virus. Apparently, efficiency of uptake of transfected synthetic RNA and cloning of mutant virus is at least as important as growth characteristics of desired mutant BTVs.

In conclusion, a targeted single-gene modification system for BTV was successfully developed without use of positive selection for rescued reassortants or desired (mutant) viruses. This method is also applicable for more detailed genetic modification of BTV to study functions of viral proteins. In addition but not proven here, the method could also be successful to incorporate more than one genome segment, like genome segments S2 and S6 encoding together the complete outer shell of BTV. Finally, for other serogroups of the genus *Orbivirus *for which reverse genetics has not been developed yet, such as Epizootic hemorrhagic disease virus, this targeted single-gene modification system method will also be applicable in order to generate mutant orbiviruses.

## Competing interests

The authors declare that they have no competing interests.

## Authors' contributions

RGPvG contributed to experimental design, performed experiments, data analysis and manuscript preparation, DV and SGPvdW carried out experiments and data analysis, PAvR initiated this project, contributed to project design, data analysis and manuscript preparation, and supervised the project. All authors read and approved the final manuscript.
